# Uncovering Regulators of Heterochromatin Mediated Silencing Using a Zebrafish Transgenic Reporter

**DOI:** 10.3389/fcell.2022.832461

**Published:** 2022-03-07

**Authors:** Audrey E. Calvird, Morgan N. Broniec, Katherine L. Duval, Alysha N. Higgs, Vani Arora, Lauren N. Ha, Erik B. Schouten, Annabel R. Crippen, Maura McGrail, Kathrin Laue, Mary G. Goll

**Affiliations:** ^1^ Department of Genetics, University of Georgia, Athens, GA, United States; ^2^ Department of Genetics, Development and Cell Biology, Iowa State University, Ames, IA, United States; ^3^ Department of Human Molecular Genetics and Biochemistry, Faculty of Medicine, Tel Aviv University, Tel Aviv, Israel

**Keywords:** heterochromatin, zebrafish, NSD1, transgene, H3K36

## Abstract

Heterochromatin formation and maintenance is critical for the repression of transcription from repetitive sequences. However, *in vivo* tools for monitoring heterochromatin mediated repression of repeats in the context of vertebrate development have been lacking. Here we demonstrate that a large concatemeric transgene integration containing the dsRed fluorescent reporter under the control of a ubiquitous promoter recapitulates molecular hallmarks of heterochromatic silencing, and that expression from the transgene array can be reactivated by depletion of known regulators of heterochromatin. We then use this reporter to identify a previously unappreciated role for the zebrafish NSD1 orthologs, Nsd1a and Nsd1b, in promoting heterochromatin mediated repression. Our results provide proof-principle that this transgenic reporter line can be used to rapidly identify genes with potential roles in heterochromatic silencing in the context of a live, vertebrate organism.

## Introduction

Fractionation of eukaryotic genomes into regions of open euchromatin and condensed heterochromatin is fundamental to genome organization, with euchromatic regions favoring transcription and heterochromatic regions promoting a repressive state (Penagos-Puig and Furlan-Magaril 2020). Heterochromatin falls on a spectrum ranging from the more labile facultative heterochromatin often found in genic regions, to more deeply repressive constitutive heterochromatin. Both dispersed repetitive elements such as transposons and large blocks of concatemeric sequence including pericentromeric repeats are typically silenced by constitutive heterochromatin (Allshire and Madhani 2018). However, differences in the precise mechanisms that drive silencing at distinct repeat classes and in different species have been noted (Saksouk, Simboeck, and Dejardin 2015).

Much of our foundational knowledge regarding the mechanisms of heterochromatin formation comes from genetic screens performed in yeast, plants and *Drosophila melanogaste*r (Vongs et al., 1993; Hollick and Chandler 2001; Schotta et al., 2003; Grewal and Jia 2007). For example, classic position effect variegation screens designed to monitor the spread of heterochromatin from *Drosophila* pericentromeres to the juxtaposed *white* gene identified core components of constitutive heterochromatin including the histone H3 lysine 9 methyltransferase SU(VAR)3-9 and the chromodomain containing protein HP1 (Reuter and Spierer 1992). Subsequent studies have demonstrated conserved functions for HP1 in binding methylated H3K9 residues and driving heterochromatin formation through compaction and phase separation (Maison and Almouzni 2004; Strom et al., 2017). Although absent from yeast and *Drosophila*, the modified base 5-methylcytosine also contributes to heterochromatic silencing at repetitive sequences in plants and vertebrate animals (Goll and Bestor 2005).

Early methods of transgenesis often resulted in many copies of the individual transgene unit integrating into a single genomic site, creating large concatemeric repeat arrays. Likely due to their repetitive nature, these large transgene concatemers are also often subject to heterochromatic silencing (Dorer and Henikoff 1994). In mouse, it was reported that a concatemeric array of a lacZ marked transgene acquired high levels of 5-methylcytosine and produced limited lacZ expression, while reduction of the array to a single transgene unit restored expression (Garrick et al., 1998). The potential for similar concatemers to serve as surrogate reporters of heterochromatin disruption in mouse was subsequently demonstrated in a genetic screen for dominant modifiers of expression from a GFP producing transgene concatemer. The screen identified both known and previously unappreciated modifiers of heterochromatic repression (Blewitt et al., 2005; Ashe et al., 2008; Daxinger et al., 2013). However, use of the human alpha-globin promoter to control GFP production limited analysis to erythroid cells (Preis et al., 2003).

Zebrafish offer another powerful model for exploring mechanisms of heterochromatin regulation in the context of vertebrate development. Small size and large broods make zebrafish particularly amenable to screening approaches, while larval clarity enables the use of fluorescent reporters to rapidly monitor transcription *in vivo,* over the course of early development. Previous attempts to develop reporters for monitoring transcriptional silencing in zebrafish have focused on fortuitous silencing of GAL4/UAS regulated transgenes (Duffy 2002; Halpern et al., 2008). When multiple copies are present in tandem, CpG rich UAS sequences become enriched in 5-methylcytosine and silenced (Goll et al., 2009; Akitake et al., 2011). However, likely due to the small size of the UAS (∼17 base pairs), these repeat clusters fail to recapitulate additional features found at larger blocks of constitutive heterochromatin including enrichment for H3K9 methylated histones (Goll et al., 2009; Akitake et al., 2011).

Here, we describe a large concatemeric transgene integration in zebrafish that recapitulates broader features of constitutive heterochromatin, including enrichment in H3K9me3. We demonstrate that silencing of the concatemeric reporter is dependent on known heterochromatic regulators including the zebrafish SU(VAR)3-9 ortholog, Suv39h1b. Demonstrating its utility, we then use this line to identify a previously unappreciated role for the PWWP/SET domain containing proteins Nsd1a and Nsd1b in mediating heterochromatic silencing at the transgene array and at endogenous pericentromeric repeats. Importantly, this transgenic line represents a unique tool for identifying modifiers of heterochromatin mediated repression in an intact, living vertebrate animal.

## Results

**FIGURE 1:**
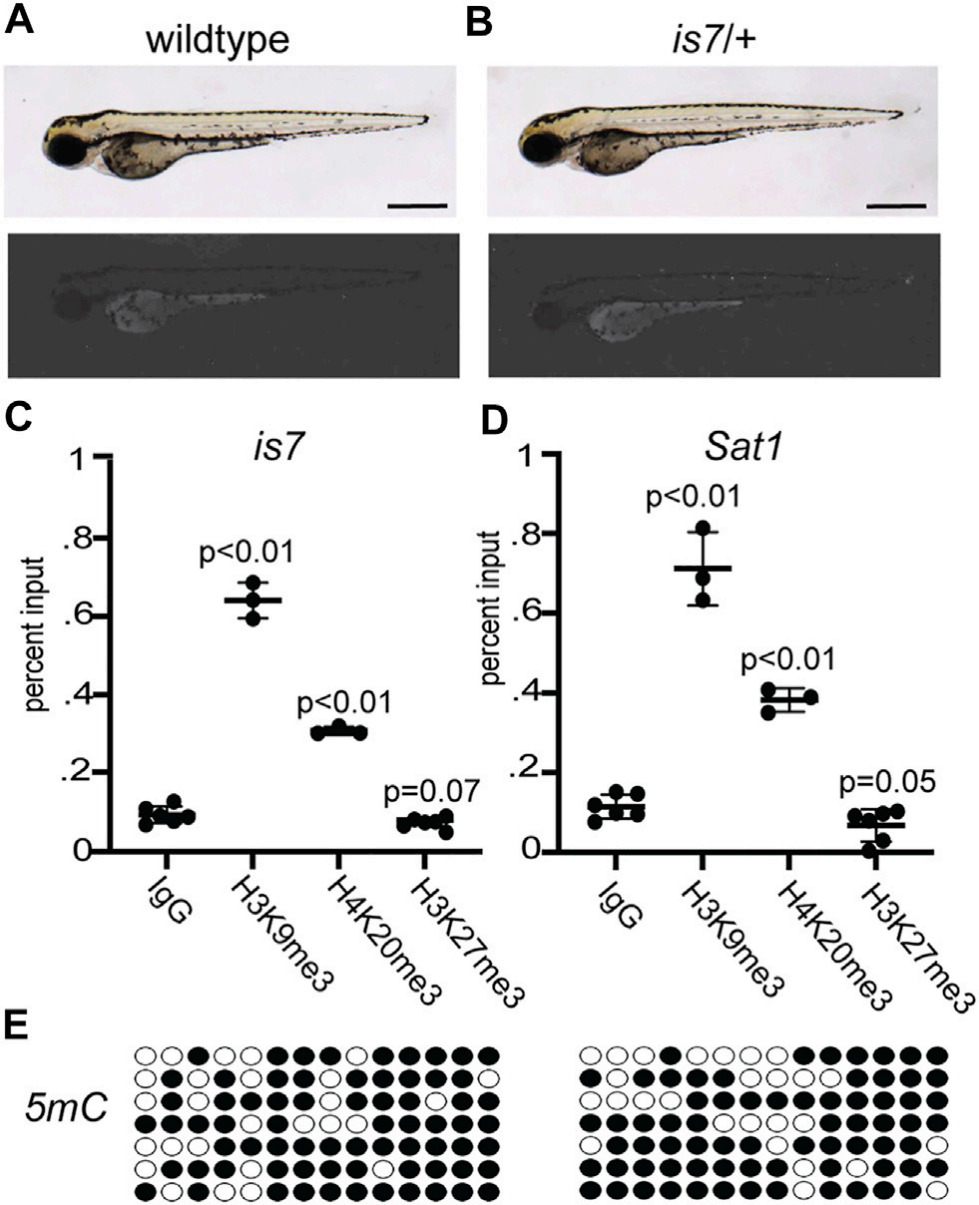
The *is7* transgene recapitulates key features of constitutive heterochromatin. **(A–B)** Brightfield (top) and fluorescence (bottom) images of representative wild-type **(A)** and *is7/+* **(B)** larva at 3 days post-fertilization (dpf). Scale bar indicates 1 mm. **(C–D)** Levels of the histone modifications H3K9me3, H4K20me3 and H3K27me3 at the *is7* transgene array **(C)** or Sat1 pericentromeric repeats **(D)** as assessed by chromatin immunoprecipitation quantitative PCR (ChIP-qPCR). **(E)** 5-methycytosine (5 mC) enrichment (filled circles) at the *is7* transgene array in two representative larval pools as detected by sodium bisulfite sequencing. Error bars indicate standard deviation.

The zebrafish *is7* transgenic line harbors a ∼70-100 copy concatemeric integration event (McGrail et al., 2011). Transgene units within the concatemer include coding sequence for the fluorophore dsRed under the control of a *bactin* promoter fragment, as well as additional non-coding DNA sequences. While the original concatemeric transgene array produced robust ubiquitous fluorescence in zebrafish larvae (McGrail et al., 2011), we noted that after breeding for more than 10 generations, zebrafish larva harboring this concatemer array produce limited dsRed fluorescence with few dsRed positive cells (Figures 1A,B). Progressive silencing of expression from the *is7* concatemer was observed across all tissues, and was reminiscent of heterochromatic silencing previously reported for repetitive transgene concatemers in other systems (Dorer and Henikoff 1994; Garrick et al., 1998). To test whether this array might undergo similar heterochromatic silencing, we compared enrichment of histone modifications at the *is7* transgene concatemer to those found at endogenous pericentromeric *Sat1* repeats, which are a known site of heterochromatic silencing in zebrafish (Laue et al., 2019; Rajshekar et al., 2018). Chromatin immunoprecipitation (ChIP) revealed that the *is7* array was enriched in histone modifications H3K9me3 and H4K20me3, which are typically associated with constitutive heterochromatin (Allshire and Madhani 2018) (Figure 1C). In contrast, H3K27me3, a modification associated with facultative heterochromatin, was not enriched at the transgene array (Figure 1C). These profiles were comparable to those observed at endogenous *Sat1* pericentromeric repeats (Figure 1D). Previous work has established that pericentromeric repeats are also heavily enriched in 5-methylcytosine in zebrafish and other vertebrate species (Goll and Bestor 2005; Rajshekar et al., 2018). Similarly, we find the *is7* transgene concatemer harbors substantial levels of this repressive epigenetic modification **(** Figure 1E). Taken together, these data demonstrate that the *is7* array recapitulates molecular hallmarks associated with heterochromatic repression.

**FIGURE 2:**
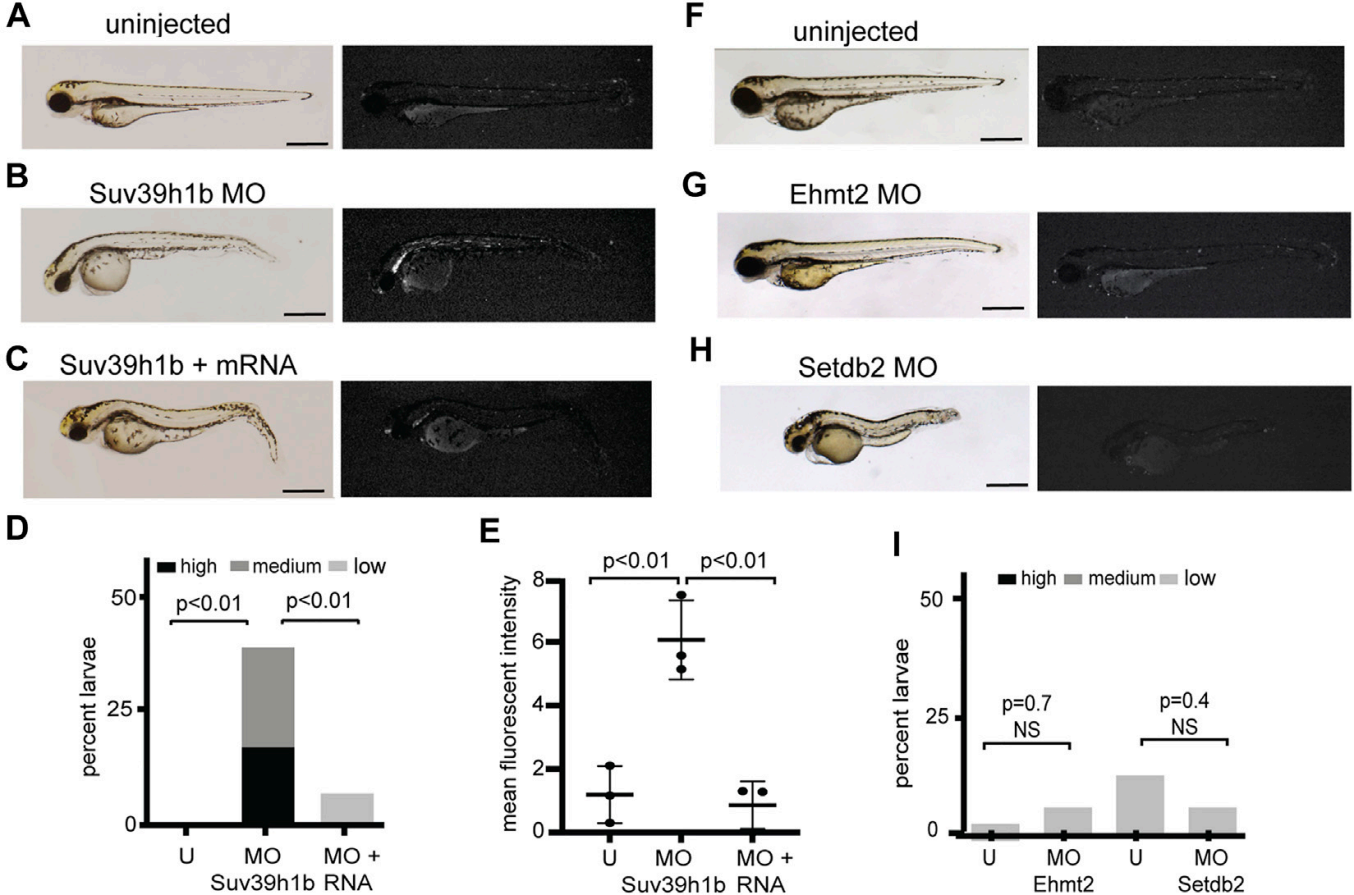
Morpholino depletion of the histone H3K9me3 methyltransferase Suv39h1b reactivates expression from the silenced *is7* transgene array. **(A–C)** Representative brightfield (left) and fluorescence (right) images of *is7*/+ transgenic larvae at 3 dpf. **(A)** Uninjected control, **(B)** larva that had been injected with 4 ng Suv39h1b morpholino at the one-cell stage, **(C)** larva that had been coinjected with Suv39h1b morpholino and 400 pg Suv39h1 mRNA (MO = morpholino). **(D)** Percent Suv39h1b morpholino injected larvae, Suv39h1b morpholino plus Suv39h1b mRNA injected larvae, and sibling uninjected control larvae that show high, medium or low dsRed fluorescence at 3 dpf (U = uninjected). Roughly 50% of larva from crosses between *is7*/+ males and wild-type females will be transgenic, therefore a maximum of ∼50% of larvae have the capacity for increased *is7* expression. *n* = 40–50 larvae per experimental condition **(E)** Mean fluorescent intensity of the three larvae showing the highest dsRed fluorescence in Suv39h1b morpholino injected larval pools, morpholino plus RNA injected larval pools and uninjected sibling control pools at 3 dpf. **(F–H)** Brightfield (left) and fluorescence (right) images of representative *is7/+* transgenic larvae that were uninjected **(F)**, injected with 4 ng of morpholino targeting Ehmt2 **(G)** or 4 ng of morpholino targeting Setdb2 **(H)** at the one-cell stage and imaged at 3 dpf. **(I)** Percent larvae showing high, medium or low dsRed fluorescence after injection with 4 ng Ehmt2 or Setdb2 morpholino compared to sibling uninjected controls. *n* = 50–60 larvae per experimental condition. All scale bars indicate 1 mm. Error bars indicate standard deviation.

To clarify whether silencing of expression from the *is7* concatemer was mediated through heterochromatic repression, we next tested whether expression could be reactivated by depletion of a known mediator of heterochromatic silencing. Orthologs of the histone H3 lysine 9 methyltransferase SU(VAR)3-9 are primary mediators of H3K9me3 establishment at highly repetitive sequences in many species, and depletion results in elevated expression from these sequences (Peters et al., 2001; Schotta et al., 2002; Rai et al., 2006). The zebrafish genome encodes for two SU(VAR)3-9 orthologs, with *suv39h1b* being predominantly expressed during the bulk of larval development (White et al., 2017). To test whether Suv39h1b was important for silencing of the *is7* array, embryos were collected from a cross between heterozygous *is7* transgenic males and wild-type females. Pools of sibling embryos were then injected with either 4 nanograms (ng) of Suv39h1b morpholino or 4 ng of Suv39h1b morpholino plus 400 picograms (pg) of morpholino resistant Suv39h1b mRNA at the one-cell stage. Additional sibling embryos were held as uninjected controls. Groups were blinded and scored for fluorescence at 3 days post fertilization (dpf) using a 4-point scale ranging from baseline to high fluorescence (Supplementary Figure S1). In contrast to non-injected controls, we observed that nearly all Suv39h1b morpholino injected larvae harboring the transgene exhibited clear dsRed fluorescence above baseline. Moreover, co-injection of morpholino resistant mRNA encoding wild-type Suv39h1b rescued array silencing (Figures 2A–D). Molecular analysis of bulk histone H3K9me3 confirmed that this modification was reduced in larvae that had been injected with Suv39h1 morpholino, and chromatin immunoprecipitation confirmed corresponding reductions in H3K9me3 at the *is7* transgene array (Supplementary Figure S2).

To further assess derepression from the *is7* array, we quantified the mean fluorescent intensity for the three larvae from each experimental group that showed the highest fluorescence. Again, significant increases in fluorescence were observed in morpholino depleted embryos compared to controls, and these increases were rescued by co-injection of morpholino resistant Suv39h1b mRNA (Figure 2E). Increased dsRed was not observed following injection of morpholinos designed to deplete Ehmt2, or Setdb2, two H3K9 methyltransferases that have not been previously implicated in heterochromatic repression at endogenous concatemeric repeats (Figures 2F–I). Taken together these findings demonstrate that a key mediator of transcriptional silencing from endogenous concatemeric repeats is also required for silencing of transcription from the *is7* transgene concatemer.

**FIGURE 3:**
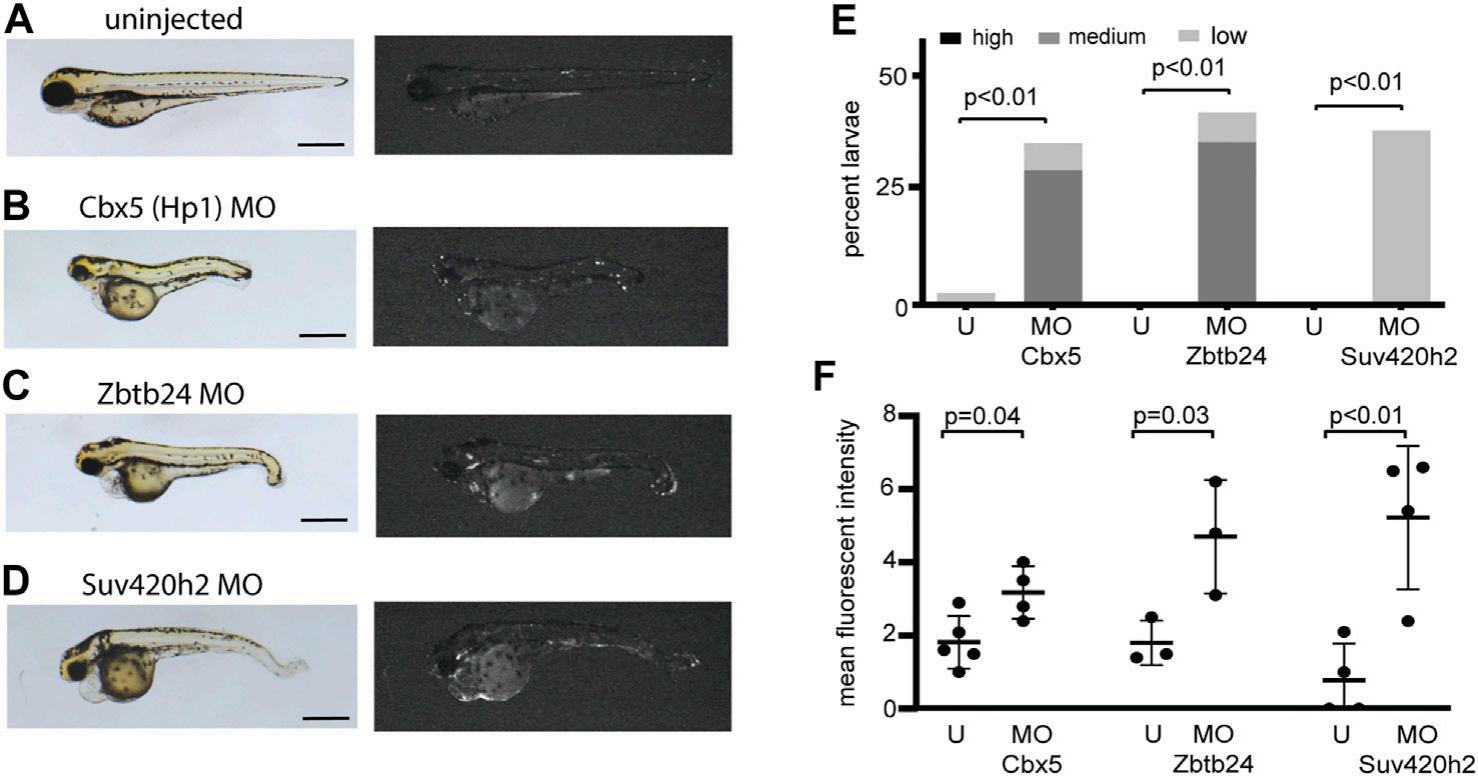
Morpholinos designed to deplete additional proteins involved in heterochromatic repression reactivate expression from the *is7* transgene array. **(A–D)** Representative brightfield (left) and fluorescence (right) images of *is7*/+ transgenic larvae at 3 dpf. **(A)** Uninjected control, **(B)** larva injected with 4 ng of Cbx5 morpholino at the one-cell stage, **(C)** larva injected with 4 ng of Zbtb24 morpholino at the one-cell stage, **(D)** larva injected with 4 ng of Suv420h2 morpholino at the one-cell stage. (MO = morpholino). **(E)** Percent larvae injected with each morpholino that show high, medium or low dsRed fluorescence at 3 dpf compared to sibling uninjected controls (U = uninjected). *n* = ∼30 embryos per condition **(F)** Mean fluorescent intensity of larvae showing the highest dsRed fluorescence in morpholino injected larval pools, compared to uninjected sibling control pools at 3 dpf. All scale bars indicate 1 mm. Error bars indicate standard deviation.

To further define genes involved in mediating transcriptional silencing from the *is7* reporter, we used morpholinos to deplete additional proteins that have been previously implicated in heterochromatic silencing at concatemeric repeats. We found that injection of a morpholino designed to deplete the zebrafish HP1 ortholog Cbx5 caused modest reactivation of the transgene (Figures 3A,B,E,F). Similarly, increased fluorescence was detected following injection of morpholinos designed to deplete Zbtb24, which is required for 5-methylcytosine mediated silencing at pericentromeric repeats, and Suv420h2, which promotes H4K20 methylation at repetitive sequences (Figures 3A,C–F). Taken together, these findings suggest that the *is7* transgene concatemer can be used as a rapid, *in vivo* tool for first pass identification of genes with potential roles in heterochromatic silencing.

**FIGURE 4:**
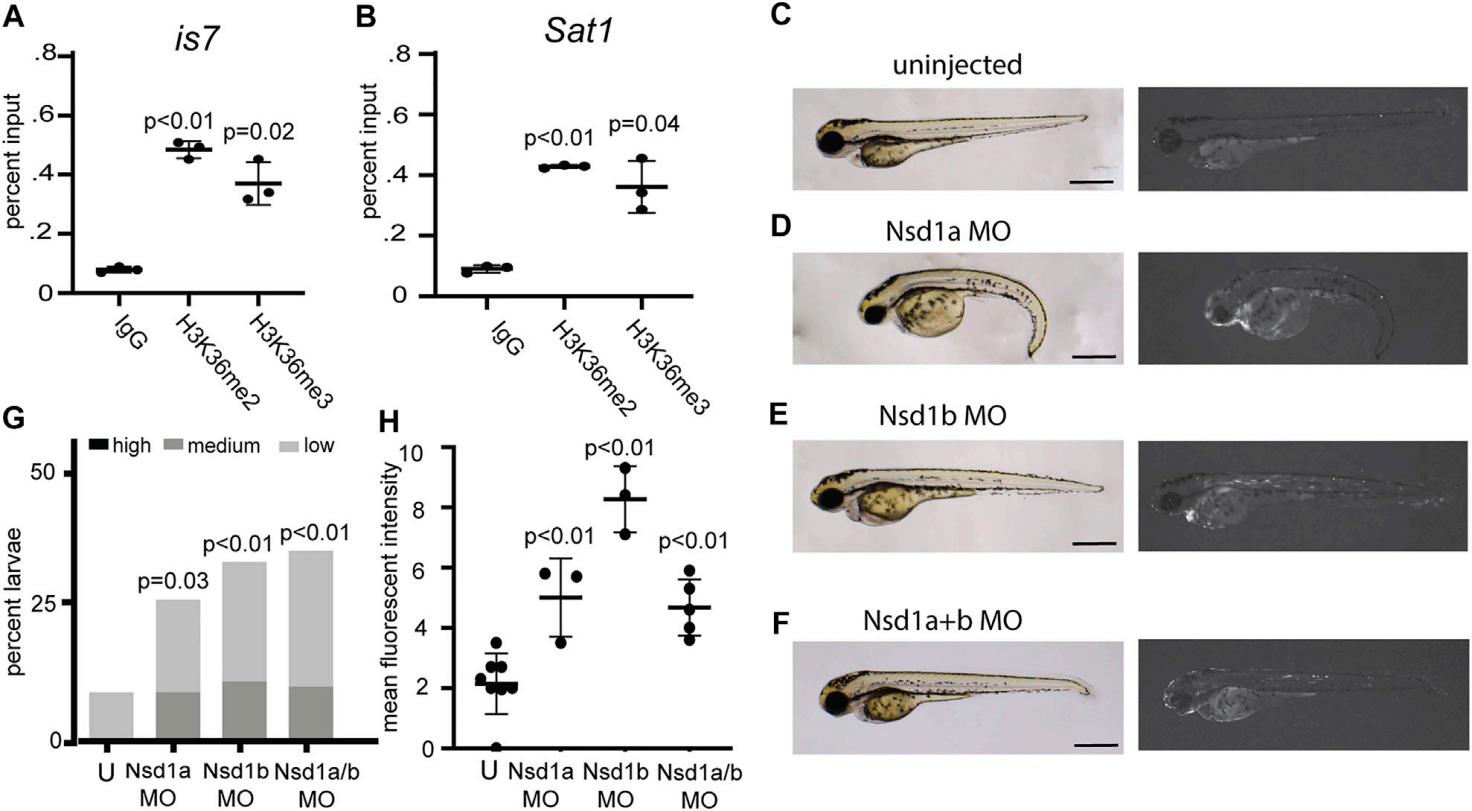
Morpholinos designed to deplete the H3K36 methyltransferases Nsd1a and Nsd1b increase expression from the *is7* transgene array. **(A–B)** Levels of the histone modifications H3K36me2 and H3K36me3 at the *is7* transgene array **(A)** and *Sat1* pericentromeric repeats **(B)** as assessed by ChIP-qPCR **(C–F)** Brightfield (left) and fluorescence (right) images of representative *is7/+* transgenic larvae at 3 dpf. **(C)** Uninjected control, **(D)** larva injected with 4 ng of Nsd1a morpholino at the one-cell stage, **(E)** larva injected with 4 ng of Nsd1b morpholino at the one-cell stage, **(F)** larva injected with 3 ng each of Nsd1a and Nsd1b morpholino at the once cell stage (MO = morpholino). **(G)** Percent larvae that show high, medium or low dsRed fluorescence at 3 dpf compared to sibling uninjected controls (U = uninjected) *n* = 50-70 embryos per condition. **(H)** Mean fluorescent intensity of larvae showing the highest dsRed fluorescence in morpholino injected larval pools, compared to uninjected sibling control pools at 3 dpf. All scale bars indicate 1 mm. Error bars indicate standard deviation.

In characterizing histone modifications at the transgene array and at endogenous *Sat1* repeats, we noted that these sequences showed some enrichment for the histone modifications H3K36me2/3 (Figures 4A,B). Previous studies have also noted H3K36 methylation at pericentromeric sequences, however its function at these sequences is unclear (Chantalat et al., 2011; Saha et al., 2020). To test whether this modification might be involved in silencing of these repeats, we individually injected morpholinos designed to deplete the H3K36 methyltransferases Nsd1a, Nsd1b, Setd2, Setmar, and Whsc1l1 into one-cell stage embryos from crosses between *is7/+* transgenic males and wild-type females. Injection of morpholinos designed to deplete Nsd1a or Nsd1b each resulted in larval pools with increased fluorescence compared to controls, while those targeting Whsc1l1, Setd2, and Setmar had no effect (Figures 4C–E,G,H; Supplementary Figure S3). Co-injection of Nsd1a and Nsd1b morpholinos resulted in similar depression (Figures 4C,F–H). These findings suggested potential roles for Nsd1 orthologs in mediating repression at repetitive sequences.

**FIGURE 5:**
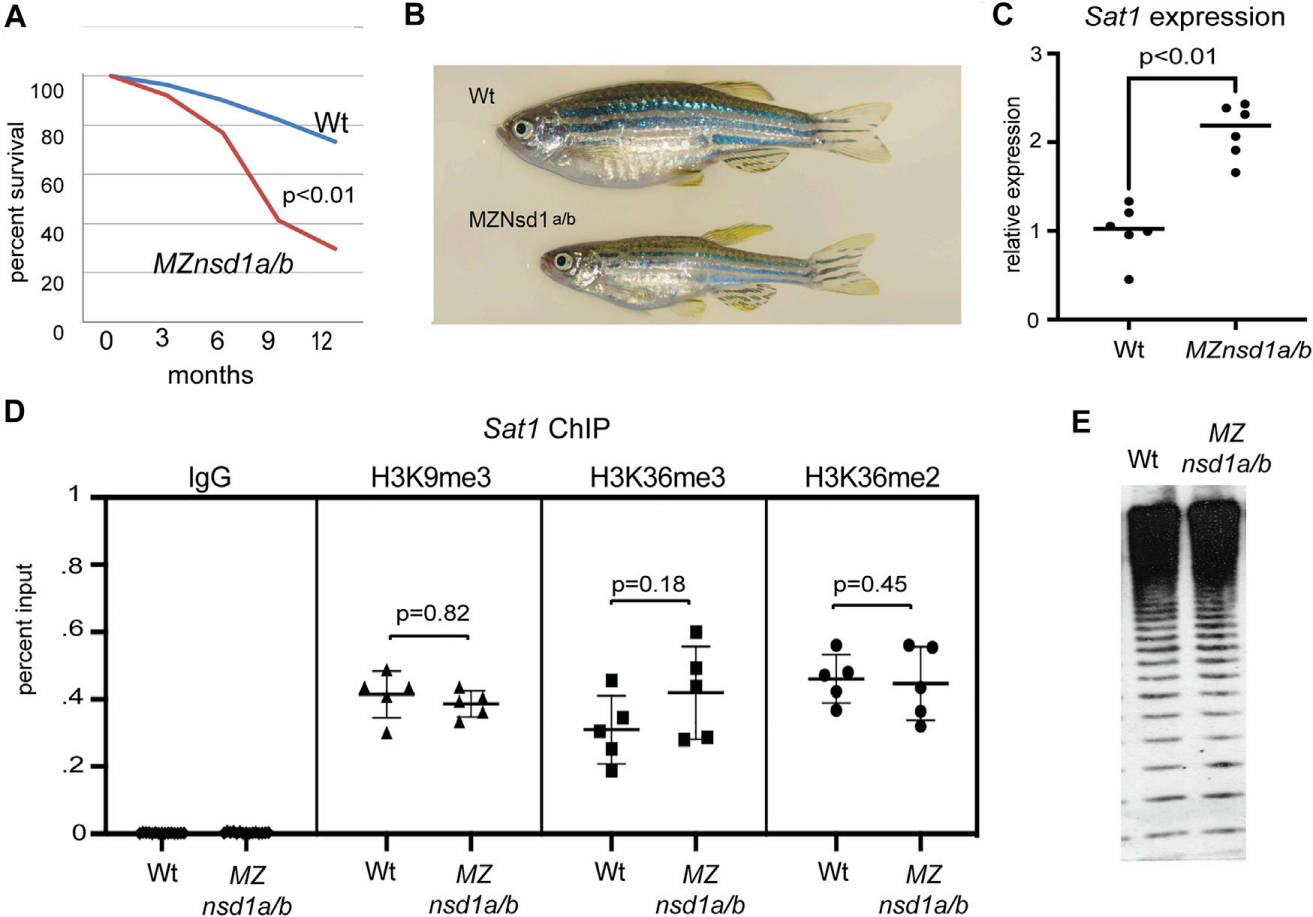
Combined homozygous deletion of *nsd1a/b* causes derepression of endogenous pericentromeric *Sat1* repeats without impacting key molecular markers of heterochromatic silencing. **(A)** Survival plot comparing wild-type and *MZnsd1a/b* homozygous deletion mutants over the first 12 months of development (*n* = ∼60 animals per genotype). **(B)** Bright field image of representative wild-type and *MZnsd1a/b* homozygous mutant adult zebrafish at 6 months post fertilization. **(C)** Expression from pericentromeric *Sat1* repeats in wild-type and *MZnsd1a/b* homozygous mutant larvae at 3 dpf as assessed by qPCR. **(D)** ChIP-qPCR for IgG, H3K3me2, H3K36me3 and H3K9me3 at Sat1 pericentromeric sequences in wild-type and *MZnsd1a/b* homozygous mutant larvae at 3 dpf. **(E)** Southern blot of genomic DNA isolated from wild-type and *MZnsd1a/b* homozygous mutant larvae at 3 dpf, digested with the methylation sensitive restriction enzyme HpyCH4IV and probed with *Sat1* sequence. Equivalent digestion suggests 5 mC levels at *Sat1* repeats are unaffected in *MZnsd1a/b* homozygous mutant larvae. (Wt = Wildtype). Error bars indicate standard deviation.

To confirm potential functions for NSD1 orthologs in promoting heterochromatin mediated repression, we validated these findings in mutants. Given the potential for transcriptional compensation among closely related genes, we chose to focus on *nsd1a/b* double mutants for this analysis. CRISPR/Cas9 was used to generate large deletions which eliminated the bulk of coding sequence for *nsd1a* and *nsd1b* (Supplementary Figure S4). We then generated *nsd1a* ^*–/–*^ *; nsd1b* ^*–/–*^ maternal/zygotic double mutants (*MZnsd1a/b*) that completely lack NSD1 orthologs by crossing. We found that *MZnsd1a/b* mutants were viable to adulthood, however they exhibited reduced survivorship, impaired growth and diminished fertility compared to age-matched wildtype controls (Figures 5A,B; Supplementary Figure S5). Similar phenotypes have been previously observed in other mutants with defects in heterochromatic silencing (Peters et al., 2001).

Consistent with a role in heterochromatic silencing, we also observed increased transcript levels from endogenous *Sat1* pericentromeric repeats in *MZnsd1a/b* mutants by reverse transcription qPCR (Figure 5C). This finding confirms preliminary results from the *is7* transgene assay, demonstrating a requirement for Nsd1 orthologs in dampening transcription from concatemeric repetitive sequence. Although we observed derepression of *Sat1* transcripts in *MZnsd1a/b* mutants, enrichment for H3K36me2/3 at pericentromeric repeats was not affected in *MZnsd1a/b* mutants, nor did we observe changes in the canonical heterochromatic markers H3K9me3 or 5-methylcytosine at pericentromeres (Figures 5D,E). These findings suggest that NSD1 orthologs may mediate repression through a mechanism that is independent of these chromatin marks.

## Discussion

Taken together our results establish the *is7* transgene array as a powerful reporter system for first pass detection of candidate genes with potential roles in heterochromatic repression, and identify previously unappreciated roles for NSD1 orthologs in mediating heterochromatic silencing at concatemeric repeats. We find that the is7 array harbors known molecular markers of heterochromatin and its expression is reactivated following depletion of known heterochromatic regulators. The patchy reactivation of the is7 array we observe is consistent with variegated reactivation observed when employing concatemeric reporter systems in other models, and likely reflects partial destabilization of heterochromatic repression across many repeat units (Dorer and Henikoff 1994; Schotta et al., 2003; Blewitt et al., 2005).

Importantly, derepression of endogenous *Sat1* sequences in *MZnsd1a/b* mutants confirms our initial findings employing the *is7* reporter line and Nsd1a/b morpholinos for rapid screening. Although we did not detect obvious changes in histone modifications or 5-methylcytosine at pericentromeric repeats in *MZnsd1a/b* mutants, clear increases in *Sat1* transcription in these mutants demonstrates that Nsd1a/b are also important for mediating repression of endogenous concatemeric repeats. In addition to harboring a SET domain capable of methylating H3K36 residues, Nsd1a and Nsd1b also contain PWWP domains capable of binding methylated H3K36, raising the possibility that Nsd1a/b could act by recognizing this modification and recruiting additional repressive machinery to these sequences (Wagner and Carpenter 2012). Alternatively, our results cannot rule out the possibility that histone modifications were impacted at only a small subset of repeats, which would be difficult to detect by chromatin immunoprecipitation, or that derepression is indirect, resulting from misregulation of additional genes that may be responsible for interpretation of these marks.

Regardless of mechanism, identification of NSD1 orthologs as candidate regulators of heterochromatin using the *is7* transgene array provides proof-principle that this transgenic line can be used to rapidly identify genes with potential roles in heterochromatic silencing in the context of a live, developing vertebrate organism. Validation of the silenced *is7* transgenic line for monitoring heterochromatic repression offers potential for large scale screens to identify additional regulators of heterochromatin maintenance and establishment in a vertebrate system. Moreover, the *is7* transgenic reporter line has the capacity for broad use in probing heterochromatin impact following embryonic exposure to environmental toxicants and other stressors. Ultimately, the unique opportunity for rapid, *in vivo* exploration of heterochromatin regulation afforded by this reporter is expected to help elucidate the complex mechanisms that support transcriptional silencing in the context of vertebrate development.

## Materials and Methods

### Zebrafish Husbandry

Zebrafish husbandry and care were in accordance with the American Association for Laboratory Animal Science as directed by the international animal conduct and use committee members at the University of Georgia. Zebrafish were raised and cared for under standard conditions in compliance with all current protocols and ethical standards. AB strain zebrafish were bred and fertile embryos were raised at 28°C. Generation of the *Tg(T2/OncZ, ß-actin:RFP)* ^*is7*^ transgenic line, referred to in this report simply as *is7,* was previously described (McGrail et al., 2011). Since the time of its initial publication, the line has been propagated for >10 generations.

### Morpholino Microinjection and mRNA Rescue

All morpholinos used in this study are listed in Supplementary Table S1. For each morpholino, 4 ng was injected into pools of ∼50 embryos at the one-cell stage. For co-injection experiments, 3 ng of each morpholino was injected for a total of 6 ng. For mRNA rescue experiments, 4 ng of morpholino was co-injected with 400 pg of *in vitro* transcribed morpholino resistant mRNA.

### Larval Screening, Image Capture and Quantification

For each condition, pools of morpholino injected and uninjected sibling larvae were screened for dsRed fluorescence at 3 days post-fertilization. In all cases, control and morphant dishes were blinded prior to screening. Following blinding, the number of larvae showing fluorescence at typical baseline levels for the silenced transgene or with low, medium or high increases in fluorescence above typical baseline were recorded (see Supplementary Figure S1). Autofluorescence was excluded as a source of signal by examining fluorescence in the GFP channel. After screening, images of the 3-5 of the larvae showing the highest fluorescence in each dish were captured for documentation and quantification. Images were captured using an Olympus MVX fluorescent Microscope with a DP72 camera and Sens Standard software (Olympus v1.13). All pictures were all taken at ×2 magnification. Brightfield images were taken with a standard exposure time of 25 ms, and RFP images were taken with a standard exposure time of 500 ms. Background subtracted mean fluorescent intensity was quantified over a hand selected area corresponding to the entire zebrafish larva using ImageJ (Schneider, Rasband, and Eliceiri 2012).

### RNA Extraction and Reverse Transcription qPCR

RNA was isolated from pools of ∼10 larvae using Trizol (Thermo Fisher 15596018). Extracted RNA was resuspended in 50 μL of nuclease free water and stored at −80 °C. RNA samples were cleared of trace DNA using TURBO DNase (Thermo Fisher AM2238) and cDNA was produced using GOSCRIPT Reverse Transcriptase Kit (Promega A5001). All qPCR experiments are performed with the Quant Studio 3 qPCR machine (Thermo Fisher). Analysis was performed using the 2^–ΔΔCt^ method, with relative mRNA levels of all transcripts normalized to β-actin1. All primer sequences are listed in Supplementary Table S3 **.**

### Chromatin Immunoprecipitation and Real-Time qPCR

ChIP was performed generally as previously described (Lindeman et al., 2009). Chromatin was sheared at 4°C using a bioruptor for 15 min at high power with 30 s on/30 s off and monitored using a 1% agarose gel. In order to provide standardized input for each ChIP experiment, chromatin was diluted to A260 = 0.2. For each ChIP, 2 μg antibody per 10 μL Dynabeads and 100 μL diluted chromatin was incubated for 8 h at 4°C. Antibodies used in this study are listed in Supplementary Table S2. After elution, ChIP DNA and input controls were purified using QIAquick PCR purification kit (Qiagen). Eluted DNA was analyzed by qPCR using primers targeting *Sat1* or *is7* listed in Supplementary Table S3.

### Methylation Analysis

Pericentromeric methylation was analyzed through a methylation sensitive Southern blot. DNA samples were first digested using the methylation sensitive restriction enzyme HpyCH4IV overnight at 37°C. Samples were then loaded into a 0.9% agarose gel and subsequently transferred to a charged nylon membrane (GVS 1226558). The *Sat1* probe was PCR amplified as previously described (Rajshekar et al., 2018), labeled using the Chemiluminescent Nucleic Acid Detection Module (Thermo Scientific 89880) and visualized using a Biorad Chemidoc Imaging System. For methylation analysis at the *is7* transgene concatemer, up to 200 ng of DNA was subject to sodium bisulfite conversion using the EZ DNA methylation-Direct Kit (Zymo Research). Bisulfite primers used for PCR amplification of the *is7* are included in Supplementary Table S3. PCR products for sequencing were cloned using the pGEM-T Easy Vector System (Promega). Sequenced reads were analyzed using the QUMA web-based interface.

### Western Blot

24 hpf embryos were homogenized in SDS sample buffer (0.63 ml 1 M Tris-HCl, pH 6.8, 1.0 ml glycerol, 0.5 ml B-mercaptoethanol, 1.75 ml 20% SDS, 6.12 ml H*2*O) and boiled for 10 min. Samples were then run on a 4–20% acrylamide gel and transferred to membrane. The membrane was split and probed with antibodies for H3K9me3 (Abcam ab8898, 1:1000) and α-tubulin (Sigma T6074, 1:1000), followed by HRP goat anti-mouse IgG (Thermofisher 32430, 1:500) and HRP Goat anti-Rabbit IgG (Active Motif 15015, 1:30,000). Signals were visualized with a BioRad ChemiDoc Imaging System.

### Statistical Analysis

All p values for qPCR, ChIP and mean fluorescent intensity graphs were calculated using the unpaired *t* Test. For embryo pools, p values were calculated using the fisher exact test with a 2 × 2 contingency table including the number of larvae with reactivation above baseline and those lacking elevated dsRed fluorescence.

### Generation of Nsd1 Mutants

Nsd1 mutant zebrafish were generated using CRISPR-Cas technology (Liu et al., 2019). Guide RNAs were designed using CHOPCHOP (Labun et al., 2019), targeting the following sequences: *nsd1a* exon 2: GGA​TAA​CGA​CGA​GGA​TGT​CA, *nsd1a* final exon: GGG​TTT​GGA​GGA​TGA​AGA​GG; *nsd1b* exon 2: GGA​GCA​AGA​TGT​GGT​TTT​TC, *nsd1b* final exon GGA​GGT​GGT​GTG​GGT​TAA​AG. Guide RNAs targeting second and last exons of *nsd1a* or *nsd1b,* were co-injected with mRNA encoding the Cas9 endonuclease and embryos were reared to adulthood. Deletions were detected by performing PCR with primers that span the targeted deletion (Supplementary Table S3), and amplified bands were analyzed with Sanger Sequencing to identify the junction. After detection of mutations in each gene, homozygous mutants for *nsd1a* and *nsd1b* were recovered and intercrossed to generate double mutants.

## Data Availability

The original contributions presented in the study are included in the article/Supplementary Material, further inquiries can be directed to the corresponding authors.
