# Supplementary material for: Uncovering Regulators of Heterochromatin Mediated Silencing Using a Zebrafish Transgenic Reporter
Source: Front Cell Dev Biol. 2022 Mar 7;10:832461. doi: 10.3389/fcell.2022.832461 (PMC8959096; doi:10.3389/fcell.2022.832461)
Supplement: Supplementary file 1 [file Table1.PDF]

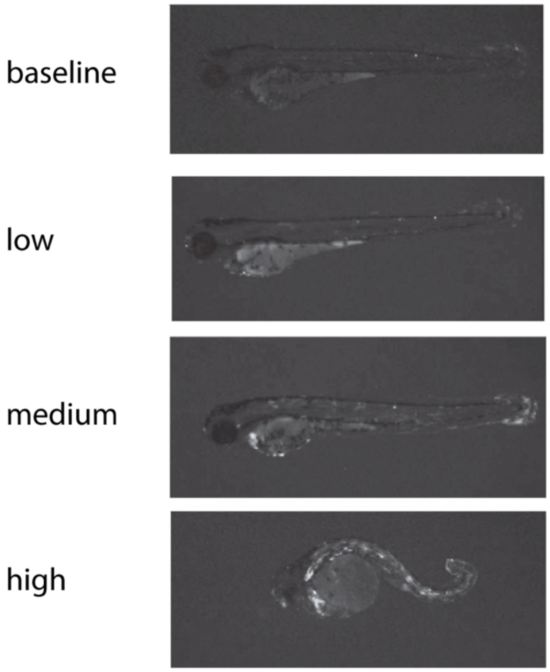

Supplementary Figure S1. Illustration of scoring system. Representative 3 dpf larvae exhibiting baseline fluorescence associated with the silenced state, and low, medium or high increases in fluorescence over baseline.

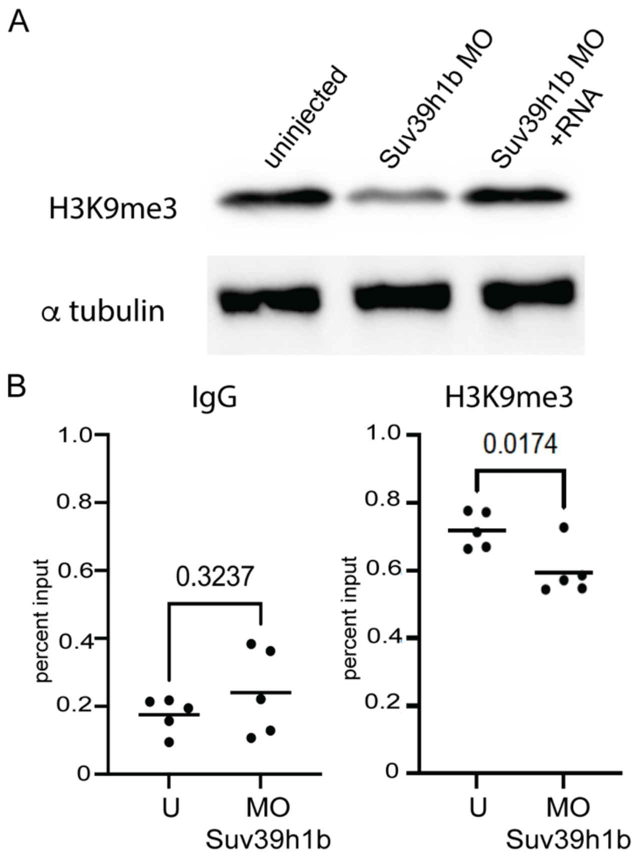

Supplementary Figure S2. H3K9me3 levels are reduced following morpholino depletion of Suv39h1b. **(A)** Western blot demonstrating reduced H3K9me3 in morpholino injected embryos compared to uninjected control or mRNA rescued embryos. α-tubulin serves as a loading control. (**B**) Chromatin immunoprecipitation demonstrates reductions in H3K9me3 levels at the transgene concatemer following morpholino depletion of Suv39h1b.

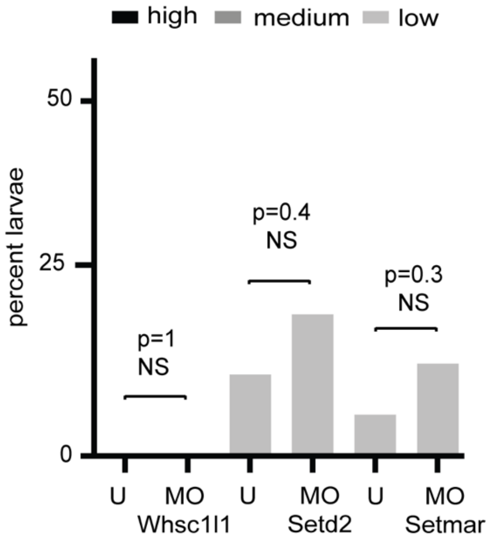

Supplementary Figure S3. Morpholinos designed to deplete the H3K36 methyltransferases Whsc1l1, Setd2 or Setmar do not cause reactivation of *is7* expression**.** Percent larvae showing high, medium or low dsRed fluorescence above baseline at 3 dpf compared to uninjected sibling larvae. For each morpholino, pools of 25-30 uninjected embryos and 25-30 morpholino injected sibling embryos were compared. (MO=morpholino, U=uninjected)

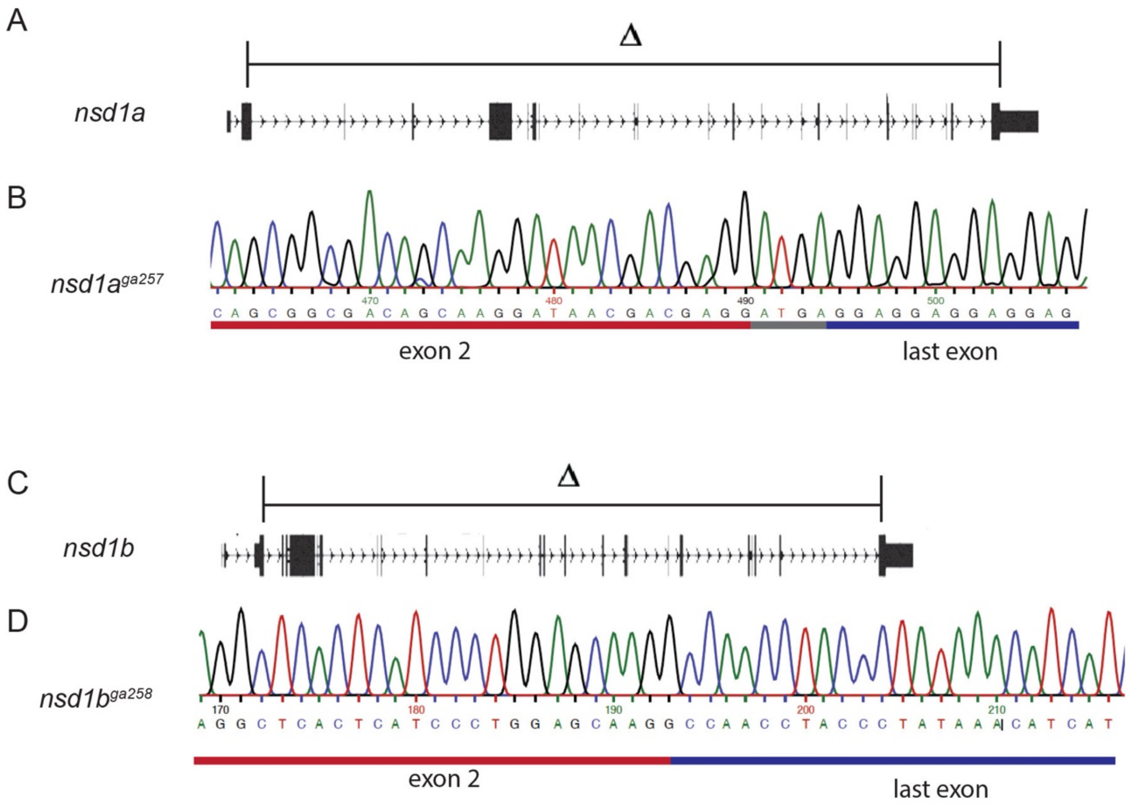

Supplementary Figure S4. Deletion of *nsd1a* and *nsd1b* genomic sequence. **(A)** Schematic indicating *nsd1a* sequence deleted in the *nsd1a*^{ga257} allele. Thick bars represent exons. **(B)** Sanger sequencing of the junction between exon 2 (red) and the final exon (blue), including a 4 base pair insertion (grey) in the *nsd1a*^{ga257} allele. **(C)** Schematic indicating *nsd1b* sequence deleted in the *nsd1b*^{gaA258} allele. **(D)** Sanger sequencing of the junction between exon 2 (red) and the final (blue) in the *nsd1b*^{ga258} allele.

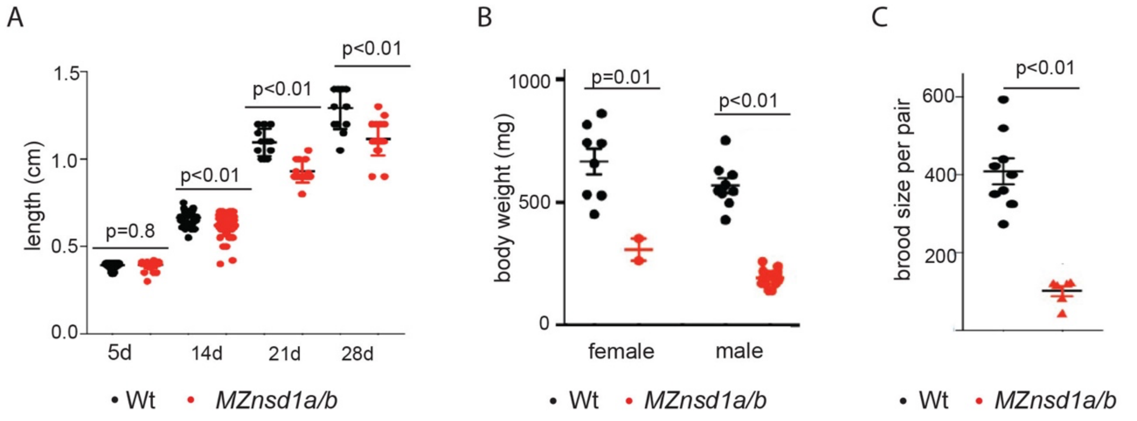

Supplementary Figure S5. *MZnsd1a/b* homozygous mutants have reduced growth and fertility. **(A)** Length of *MZnsd1a/b* homozygous mutant larvae compared to age matched wild-type controls during the first month of development (d=days, cm=centimeters, Wt=wildtype). **(B)** Weight of *MZnsd1a/b* homozygous mutants compared to age matched wild-type controls at 6 months post fertilization. (mg=milligrams) (**C**) Brood size produced from *MZnsd1a/b* homozygous mutants compared to age matched wild-type controls. Error bars indicate standard deviation.

Name	*Sequence*
Suv39h1b	CAAATTTTCCGCCATCGTCTCACGC
Ehmt2	TGATGAAAACACACACACCTTGTTG
Setdb2	CACGCACACAGATGACTGACCCTGT
Cbx5	ATGGCTGATTCTGAAAGTCTTTACT
Zbtb24	AAGGGCAGACATGATGACCTTACTC
Suv420h2	AAAGAAGTGATCCTAACCAGTGTCG
Nsd1a	CTCTGCTTGAGATTTGTCTTCACCT
Nsd1b	ATAACGATGAGTTTGACTTACCAGC
Whsc1l1	AACACGAGTAGTTCTTACCAGCAGA
Setd2	CCTGGAAGAGCTTGAAAGTACAAGA
Setmar	ACGAAAGAAGATGTGACTACCTGAA

Supplementary Table S1. Morpholinos used in this study. With the exception of Suv39h1b, Cbx5 and Zbtb24, morpholinos were previously described in (Huang et al. 2013).

Antibody target	Catalogue number
H3K9me3	ab8898
H3K27me3	ab6002
H4K20me3	ab9053
H3K36me2	ab9049
H3K36me3	ab9050
IgG	ab150085

Supplementary Table S2. Antibodies used in this study

Primer Target/Name	Sequence
SAT1F	GTCTCTGACTGAGTTTGCATTAC
SAT1R	ACATTCTGAATTGGACGTTGA
is7F	CGAGCAGGAGATGGGAACC
is7R	CAACGGAAACGCTCATTGC
SAT1ChIPF	AAGCAAGTTGCAAGTGAAAATCT
SAT1ChIPR	AGTCAGCCAGCAGAGAGGTC
is7ChIPF	CGCAGAGCTCCGAAAGTTTA
is7ChIPR	GGCAGATGATATTCCGCACT
is7bisulfiteF	TGTAAGAAGTAAGTGTAGTAATGTGTA
is7bisulfiteRa	CACCTTAAATTTAAAAAAATTTATC
nsd1adeltaF	GAAGAACCTTCACTCATCCCTGT
nsd1adeltaR	TGTTTTCCAGCGCTTTTTAGGAG
nsd1bdeltaF	GGACTCATCCAGTCCGTTTG
nsd1bdeltaR	GGCTACATCAAGGGGAGAGG

Supplementary Table S3. Primers used in this study
